# Comparison of machine learning algorithms and multiple linear regression for live weight estimation of Akkaraman lambs

**DOI:** 10.1007/s11250-024-04049-0

**Published:** 2024-09-03

**Authors:** Özge Kozaklı, Ayhan Ceyhan, Mevlüt Noyan

**Affiliations:** 1 Department of Animal Production and Technologies, Faculty of Agricultural Sciences and Technologies, Niğde Ömer Halisdemir University, Niğde, 51240 Turkey; 2 Nigde Omer Halisdemir University, Bor Vocational School, Bor/Niğde, Turkey

**Keywords:** Artificial neural network analysis, K-Fold cross validation, Live weight, Random Forest

## Abstract

This study was designed to predict the post-weaning weights of Akkaraman lambs reared on different farms using multiple linear regression and machine learning algorithms. The effect of factors the age of the dam, gender, type of lambing, enterprise, type of flock, birth weight, and weaning weight was analyzed. The data was collected from a total of 25,316 Akkaraman lambs raised at multiple farms in the Çiftlik District of Niğde province. Comparative analysis was conducted by using multiple linear regression, Random Forest, Support Vector Machines (and Support Vector Regression), Extreme Gradient Boosting (XGBoost) (and Gradient Boosting), Bayesian Regularized Neural Network, Radial Basis Function Neural Network, Classification and Regression Trees, Exhaustive Chi-squared Automatic Interaction Detection (and Chi-squared Automatic Interaction Detection), and Multivariate Adaptive Regression Splines algorithms. In this study, the test dataset was divided into five layers using the K-fold cross-validation method. The performance of models was compared using performance criteria such as Adjusted R-squared (Adj-${R}^{2}$), Root Mean Square Error (RMSE), Mean Absolute Deviation (MAD), and Mean Absolute Percentage Error (MAPE) by utilizing test populations in the predicted models. Additionally, the presence of low standard deviations for these criteria indicates the absence of an overfitting problem. ${R}^{2}$The comparison results showed the Random Forest algorithm had the best predictive performance compared to other algorithms with Adj-${R}^{2}$, RMSE, MAD, and MAPE values of 0.75, 3.683, 2.876, and 10.112, respectively. In conclusion, the results obtained through Multiple Linear Regression for the live weights of Akkaraman lambs were less accurate than the results obtained through artificial neural network analysis.

**Supplementary Information:**

The online version contains supplementary material available at 10.1007/s11250-024-04049-0.

## Introduction

Sheep farming is a significant livestock sector worldwide, providing economically valuable products such as meat, milk, and wool (FAO 2023). The live weight of sheep is an important trait in meat production (Duguma et al. 2010) and is a key in the selection programs. Therefore, accurate prediction of live weight in sheep holds great importance for sheep breeders and small farmers (Hamadani et al. 2022).

Body weight is a very important aspect of the production of sheep and lambs. It can be used to evaluate an animal’s general condition, selection or culling males and females, ration formulation in order to rapid growth, administer the correct amount of treatment, estimate market weight, and other purposes. it is necessary to know the animal’s body weight. Often farmers rely on visual judgment to determine body weight, which is not an accurate way to manage sheep. The easiest way to assess an animal’s body weight is to weigh it using a scale. However, estimating body weight accurately requires suitable equipment, which is not always available due to high cost and transportation challenges. This means that most rural livestock farmers do not have access to a scale. Therefore, there is a need for estimation of sheep body weight from simple and easily measurable variables such as linear body measurements (Younas et al. 2013; Boujenane and Halhaly 2015; Canaza-Cayo et al. 2024). However, the sheep industry the lack of technological knowledge on the part of farmers and the economic limitations of the sector have made such as precision livestock farming difficult for this technification to take place (Samperio et al. 2021). Recently, some methods have been developed to predict live weight, such as machine learning (ML) and multiple linear regression. ML algorithms can support or replace traditional methods in modeling data and making predictions or forecasts without the assumptions of traditional statistical methods (Gilbert et al. 2010). In addition, ML enables the use of complex algorithms that allow machines to learn from data and automatically make predictions without human intervention, without having to be explicitly programmed for the task (Sen et al. 2018; Gilbert et al. 2010).

Traditionally, live weights have been determined by the direct weighing of animals. However, these methods are time-consuming and labor-intensive, and sometimes it isn’t easy to obtain accurate data (Duguma et al. 2010; Hamadani and Ganai 2023; Tırınk et al. 2023b). Artificial neural network (ANN) analyses have shown notable success in modeling complex relationships and predicting nonlinear functions (Yucedag 2019; Hamadani and Ganai 2023). ANNs have been increasingly employed in various fields of livestock studies, including the prediction of genetic traits (Hamadani and Ganai 2023), animal health and disease diagnosis (Yang et al. 2000; Eksteen and Breetzke 2011; Zaborski and Grzesiak 2011), milk yield (Karadas et al. 2017), and meat quality analysis (Fukuda et al. 2013). Several algorithms are commonly used to analyse sheep live weight, including Random Forest (RF) (Huma and Iqbal 2019; Tırınk 2022), Support Vector Machines (SVM) or Support Vector Regression (SVR) (Huma and Iqbal 2019; Tırınk, 2022; Iqbal et al. 2022; Tırınk et al. 2023a, b), eXtreme Gradient Boosting (XGBoost) or Gradient Boosting (GBoost) (Faraz et al. 2023), Bayesian Regularized Neural Network (BRNN) (Tırınk 2022), Radial Basis Function Neural Networks (RBFNN) (Çelik et al. 2017), Classification and Regression Trees (CART) (Ali et al. 2015; Koc et al. 2017; Olfaz et al. 2019; Tırınk 2022; Iqbal et al. 2022; Faraz et al. 2023), Exhaustive Chi-squared Automatic Interaction Detection (Exhaustive CHAID) or Chi-squared Automatic Interaction Detection (CHAID) (Ali et al. 2015; Çelik et al. 2017; Koc et al. 2017; Olfaz et al. 2019; Faraz et al. 2023), and Multivariate Adaptive Regression Splines (MARS) (Ali et al. 2015; Çelik et al. 2017; Tırınk 2022; Tırınk et al. 2023a, b; Faraz et al. 2023). Furthermore, ANN analyses can help to achieve more accurate and rapid results, potentially enhancing the sustainability and productivity of sheep farming while facilitating inter-species differentiation (Duguma et al. 2010; Tırınk et al. 2023b).

This study aims to compare the use of multiple linear regression and artificial neural network algorithms by analyzing the weaning weights of Akkaraman lambs raised in different farms. Our study emphasizes the importance of using artificial neural network algorithms in live weight estimation of Akkaraman lambs. Moreover, the size of the data set used in our study is much larger than other studies in the literature. This increases the generalizability and reliability of our model. In addition, this study is important due to the fact that a wide variety of algorithms have been tried on a dataset. This provides the possibility to directly compare the performance of different algorithms and identify the most appropriate model. In conclusion, these findings demonstrate the potential of neural network algorithms in live weight estimation and provide a basis for future research in this field.

## Materials and methods

### Animal material and management

The study was conducted at Çiftlik district, which is located within the north-northwest borders of Niğde province of Turkey, with a latitude of “38.175053” longitude of “34.48477” and an altitude of 1555 m above sea level. The study included the Akkaraman sheep breed of Turkey. This breed has the largest population among the sheep breeds of Turkey and nearly constitutes the whole sheep population of Niğde province. Outliers dates were excluded by the standard deviation method, values that fall outside the range of Mean − 2*Standard deviation, Mean + 2*Standard deviation (Cilgin et al. 2023). The data consisted of a total of 25,316 Akkaraman lambs born to different aged mothers from 10 different farms in the Çiftlik district of Niğde province between 2006 and 2020. After lambing, the lambs were ear tagged and their birth weights were determined. In addition, information such as date of birth, type of birth, sex, and mother tag number were recorded. Colostrum intake of lambs was checked after birth. Lambs were kept in individual birth pens with their mothers for one week after birth. Lambs were given lamb starter feed and alfalfa hay from the second week after birth. The lambs were weaned at approximately 90 days of age and their live weights were weighed with a 100 g sensitive scale. In the farms, sheep were kept in the pasture most of the year and in winter they were kept in the sheepfold. Although the enterprises are different, they are generally engaged in extensive breeding. The quality of the pastures, the care and feeding of the animals and the level of knowledge and experience of the enterprises are very similar to each other.

### Multiple linear regression

The linear regression model was constructed using the R programming language and the lm() function (R Studio Team 2023). In the model, the dependent variable was the post weaning weight (Y), and the independent variable include birth weight (${X}_{1}$), weaning weight (${X}_{2}$), gender (${X}_{3}$), lambing type (${X}_{4}$), dam ages (${X}_{5}$), farmID (${X}_{6}$) and flock type (${X}_{7}$). The linear regression model can be written as:

1 $$\eqalign{& Y = {\beta _0} + {\beta _1}{X_1} + {\beta _2}{X_2} + {\beta _3}{X_3} \cr & + {\beta _4}{X_4} + {\beta _5}{X_5} + {\beta _6}{X_6} + {\beta _7}{X_7} + \cr}$$

and ${\beta }_{0}$, ${\beta }_{1}$, …, ${\beta }_{7}$ represent the regression coefficients, $\epsilon$ stands for the error term ($\epsilon \sim \left(0. {\sigma }^{2}\right)$). The assumptions for the error were examined using four different plots generated through the “Plot()” command in the R program. This command produces four distinct graphs:

1. Residuals vs. Fitted: This graph is used to evaluate the adequacy of the model’s performance. In this graph, the difference between the predicted value (fitted) and the actual value (residual) for each observation is shown. If a pattern or trend is observed in this graph, it may suggest that the model is not functioning properly.
2. Normal Q-Q: This graph is utilized to evaluate whether the model meets the assumption of normal distribution. If the observations exhibit a normal distribution, the points will align along the line.
3. Scale-Location: This graph is used to assess whether the model fulfills the assumption of homoscedasticity (constant variance). If the observations demonstrate homoscedasticity, the points will lie along a horizontal line.
4. Residuals vs. Leverage: This graph is employed to assess the influence of each observation on the model’s outcomes. If an observation possesses a high Cook’s distance value in this graph, it could significantly impact the model’s results.

In regression analysis, the Variance Inflation Factor (VIF) has been employed as a multicollinearity test to detect potential multicollinearity arising from high correlation among independent variables. The formula for VIF is defined as:

2 $$VIF = \frac{1}{1-{R}^{2}}$$

3 $${R}^{2}= 1-\frac{{\sum }_{i=1}^{n}(Yi - \widehat{Y})^{2}}{{\sum }_{i=1}^{n}(Yi - \underset{\_}{Y})^{2}}$$

Here, ${R}^{2}$ represents the proportion of variance in one independent variable that is explained by the other independent variables. If the VIF value is greater than 1, it indicates that the independent variable is highly explained by other independent variables, which can be problematic for multiple linear regression analysis.

### Artificial neural network analysis

The dataset has been randomly divided into two parts, with 75% for training and 25% for testing, as outlined by Tırınk 2022. Eight machine learning algorithms were utilized in this study, including Artificial Neural Network (ANN) using various packages such as “randomForest” for RF, “e1071” for SVM and SVR, “xgboost” for XGBoost and GBoost, “brnn” for BRNN, “RSNNS” for RBRNN, “rpart” for CART, and “party” for Exhaustive CHAID, CHAID, and “earth” for MARS algorithms, all within the RStudio environment (R Studio Team 2023).

The Random Forest algorithm, as proposed by Breiman, is developed based on the methods he recommended. Random Forest (RF) constitutes a group of learning methods that can be applied to regression, classification, and other tasks. During the training phase, it generates a considerable number of decision trees and subsequently operates by establishing the class, which entails either the average prediction (regression) or the model of classes (classification), for each individual tree (Huma and Iqbal 2019).

The Support Vector Machine (SVM) method is an algorithm developed based on the principles of statistical learning theory and introduced by Vapnik. SVM constructs a binary classifier by creating a linear separation hyperplane to classify data samples (Vapnik et al. 1996; Vapnik 2000). Its primary objective is to find a hyperplane that best distinguishes data from different classes. On the other hand, Support Vector Regression (SVR) is a variation of SVM that adapts its classification purpose to regression problems. SVR aims to arrange data points around a hyperplane. In other words, while SVR constructs a hyperplane to predict data, it focuses on regression problems rather than classification. SVM and SVR fundamentally share the same principles, such as support vectors, separation hyperplanes, and minimizing marginal errors. However, while SVM is used for classification, SVR is employed to solve regression problems. In essence, both methods stem from the same fundamental idea but are tailored variations for distinct problems (Tırınk et al. 2023b).

The eXtreme Gradient Boosting (XGBoost) algorithm was initially proposed by Chen and Guestrin (2016). XGBoost, an improved version of the GBoost algorithm (Friedman 2001), employs decision trees to define groups and leverages effective independent variables during the training process. Serving as an enhanced iteration of the GBoost algorithm, XGBoost’s fundamental objective is to create decision trees with high variance and low bias. This algorithm capitalizes on utilizing influential independent variables during its training phase (Friedman 2001; Coşkun et al. 2023).

The Bayesian Regularized Neural Network (BRNN) algorithm can generate genetic models that encompass both additive and dominant effects while making use of explicit parallel processing from multi-core architectures. BRNN can also address overfitting issues by employing Bayesian regularization methods, contributing to the model’s enhanced consistency and accuracy in delivering results (Pérez-Rodríguez et al. 2013).

Radial Basis Function Neural Networks (RBFNN), Classification and Regression Trees (CART), and Exhaustive Chi-Squared Automatic Interaction Detection (CHAID) are tree-based algorithms that use multi-way splitting to form homogeneous subsets based on Bonferroni adjustment (Ali et al. 2015). These three-stage data mining algorithms, which analyze both qualitative and quantitative data, recursively partition and merge data until the differences between the predicted and actual values are minimal. CHAID was introduced by Kass (1980), while Biggs et al. (1991), proposed Exhaustive CHAID. CART, a regression tree algorithm introduced by Breiman (2017), recursively splits the data into homogeneous subsets until the minimum difference is reached (Fukuda et al. 2013). CHAID and Exhaustive CHAID use multi-way node splitting, while CART exclusively employs binary node splitting (Breiman 2017). The Bonferroni method is used to determine adjusted significance values for merging and splitting criteria. The minimum tree depths are set as CHAID (3), Exhaustive CHAID (3), and CART (5) algorithms by default Ali et al. (2015).

The Radial Basis Function Neural Networks (RBFNN) algorithm is a type of feedforward neural network with only one hidden layer and is known for its faster learning capability compared to other feedforward networks. This advantage translates into shorter computation times when compared to other algorithms (Erol et al. 2008). The activation function of the network is chosen as the Gaussian function, and the objective function is selected as the Least Squares (LS) criterion (LeCun et al. 2015).

The Multivariate Adaptive Regression Splines (MARS) algorithm was proposed by Friedman (1991), to address classification and regression-type problems. The MARS algorithm constitutes a regression procedure that efficiently explains interactions between explanatory and response variables, as well as linear and nonlinear effects, offering an effective means of explanation (Friedman 1991).

### Model Comparison Metrics

To evaluate each model’s performance in a 5-fold cross validation framework, in the comparative analysis of machine learning algorithms and regression models, adjusted coefficient of determination (${R}^{2}$) and prediction performance metrics such as root mean square error (RMSE), mean absolute deviation (MAD), and mean absolute percentage error (MAPE) have been employed (Zhang and Hu 1998; Yucedag 2019). These metrics provide information about the model’s fitting and predictive abilities; a model with high ${R}^{2}$ (Eq. 3) and low RMSE, MAD, and MAPE values are considered more successful (Zhang and Hu 1998; Eyduran et al. 2019; Zaborski et al. 2019). The equations for these criteria are as follows:

4 $$RMSE = \sqrt{\frac{{\sum }_{i=1}^{n}(Yi - \widehat{Y}i)^{2}}{n} }$$

5 $$MAD=\frac{{\sum }_{i=1}^{n}\left|Yi - Yi\right|}{n}$$

6 $$MAPE=\frac{{\sum }_{i=1}^{n}\left|\frac{Yi - \widehat{Y}i}{Yi}\right|}{n}\times 100, \left(Yi\ne 0\right)$$

7 $${aAdj-R}^{2}=1-\left(1-{R}^{2}\right)\frac{\left(n-1\right)}{\left(n-p-1\right)}$$

In these equations, $Yi$ represents the actual values, $\widehat{Y}i$ denotes the predicted values, and p represents the number of independent variables, while n denotes the number of observations.

## Results

For the prediction of the dependent variable, which is the post weaning weight, explanatory variables consisting of both categorical and continuous have been used. The categorical explanatory variables include gender, lambing type, dam ages, enterprises, and type of flock. To explore the correlations between categorical explanatory variables and the dependent variable, Spearman’s rank correlation coefficient has been utilized. The continuous explanatory variables, weaning weight and birth weight, have been investigated for their correlations with the dependent variable using the Pearson correlation coefficient. Some descriptive statistics and correlation coefficients for these variables are presented in Table 1.

**Fig. 1:**
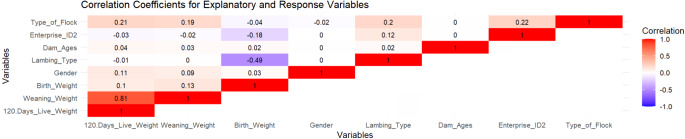
The correlation coefficient for explanatory and response variables

Figure 1 shows that weaning weight is strongly correlated with the post-weaning weight of lambs. Additionally, gender differences were found to have an impact of around 10% on this measurement in the present study. This trend is also evident in the farm effect category.

**Table 1:** Descriptive statistics for quantitative variables

Numeric Variables Names	Mean	Sd	Min	Max
Birth weight	4.084	0.854	1.500	6.500
Weaning weight	23.221	6.291	12.000	40.000
Post-weaning weight	29.646	7.106	15.000	54.418

The regression model was estimated to investigate the linear relationship between the variables associated with the post-weaning weights and the dependent variable. The obtained results of the estimated regression model are presented in Table 2. Prior to examining the results of the predicted regression model, its validity was assessed. The assumptions of error terms and the absence of multicollinearity among variables were checked for the validity of the regression model presented in Table 2.

**Table 2:** Evaluation of validity of multiple linear models used to predict the post-weaning weight of Akkaraman lambs

Coefficients	Parameter estimates			Model Inf.	
	$\beta$	SE($\beta$)	*p*-value	Model	sd
Intercept	9.106	0.299	0.000	RSE	4.426
Birth weight	−0.274	0.043	0.000	R^2^	0.612
Weaning weight	0.859	0.005	0.000	Adj R^2^	0.612
Gender of lamb	0.622	0.062	0.000	F-Stats	4562
Type of lambing	−0.860	0.101	0.000	F-Stats P-value	0.000
Ages of dam	0.105	0.021	0.000		
Enterprises	−0.087	0.012	0.000		
Type of flock	1.533	0.086	0.000		

**Fig. 2:**
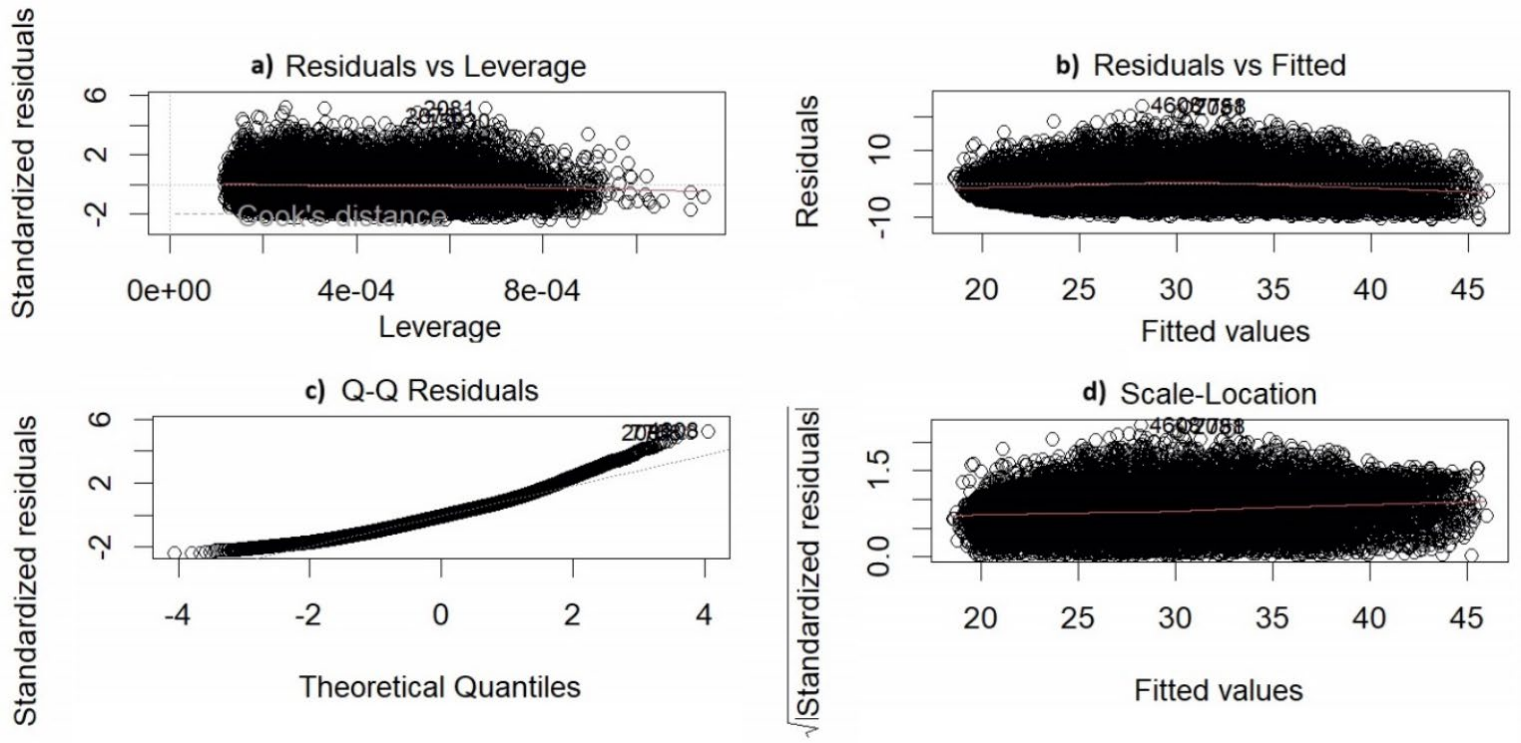
Graphs Utilized for Error Term Validation in Linear Model Validity. (a: Graph of Residuals vs. Fitted, b: Graph of Normal Q-Q, c: Graph of Scale-Location, d: Graph of Residuals vs. Leverage)

The assumptions regarding the error terms are given in Fig. 1. The VIF values were calculated as follows: 1.386338 for the birth weight variable, 1.074707 for the weaning weight variable, 1.010778 for the gender variable, 1.383125 for the lambing type of variable, 1.003036 for the dam ages variable, 1.089101 for the farm variable, and 1.145467 for the type of flock variable. In this study, since VIF values were found to be less than 5, there was no significant multicollinearity problem between independent variables. The validity of the model was visually tested using the graphs provided in Fig. 2 for the assumptions of error terms. The current graphics in residuals show that there is no problem with changing variance (a), the assumption of normal distribution is met (b), residuals are distributed with constant variance (c), and residuals are not affected by independent variables (d).

Thus, the multiple linear regression model summarized in Table 2 was found to be valid for post-weaning body weight estimation. However, interpreting the estimated coefficient and intercept for farm numbers in the accepted model is not appropriate. When keeping other independent variables constant in the model except for the “birth weight” variable, a 1-unit increase in birth weight implies a decrease of 0.224 units in the " post-weaning weight” variable. It is generally observed that the influence of birth weight on live weight decreases as age progresses. Holding other independent variables constant, a 1-unit increase in weaning weight results in a 0.883-unit increase in the " post-weaning weight " variable. For categorical variables, having a male lamb increases the live weight by 0.55 units.

Considering the coefficient for the “Lambing type” variable, the occurrence of multiple births results in a decrease of 0.74 units in the post-weaning weight. According to the model, an increase of 1 unit in the age of ewes leads to a 0.084 unit increase in post-weaning weight. Animals with an elite flock type have a live weight that is 1.366 units higher on the post-weaning weight compared to those with a base flock type.

The study conducted multiple trials to determine the best topology for the algorithms used. Various models with different numbers of hidden layers (ranging from one to five) were tested, along with testing the number of neurons in each hidden layer individually. Based on the results of these trials, the optimal network was determined to be the one with the highest fit and lowest error rate.

The calculated metrics for assessing the performance of machine learning models and multiple linear regression are presented in Table 3. For a visual representation of the model performances, Fig. 2 can be examined.

**Table 3:** Comparison criteria of (K-fold 5) models used in post weaning weight estimation in Akkaraman lambs

Models/Criteria	${R}^{2}$	$Adj-{R}^{2}$	RMSE	MAD	MAPE	Predicted BW (kg)
Multi-Linear Regression	0.611 ± 0.004	0.611 ± 0.004	4.428 ± 0.038	3.455 ± 0.036	11.992 ± 0.143	29.207
RF	0.753 ± 0.007	0.752 ± 0.007	3.683 ± 0.047	2.876 ± 0.035	10.112 ± 0.125	29.139
SVM and SVR	0.647 ± 0.008	0.646 ± 0.008	4.239 ± 0.081	3.223 ± 0.042	10.999 ± 0.139	28.773
XGBoost and GBoost	0.634 ± 0.005	0.653 ± 0.005	4.271 ± 0.024	3.270 ± 0.017	11.006 ± 0.078	28.289
BRNN	0.620 ± 0.007	0.620 ± 0.007	4.382 ± 0.041	3.414 ± 0.026	11.830 ± 0.100	29.115
RBFNN	0.596 ± 0.003	0.595 ± 0.003	4.518 ± 0.486	3.518 ± 0.035	12.198 ± 0.124	29.126
CART	0.587 ± 0.011	0.586 ± 0.011	4.569 ± 0.057	3.563 ± 0.048	12.397 ± 0.184	29.079
Exhaustive CHAID and CHAID	0.618 ± 0.008	0.617 ± 0.012	4.391 ± 0.050	3.410 ± 0.032	11.807 ± 0.161	29.111
MARS	0.628 ± 0.003	0.627 ± 0.002	4.335 ± 0.012	3.375 ± 0.009	11.690 ± 0.044	29.115

## Discussions

**Fig. 3:**
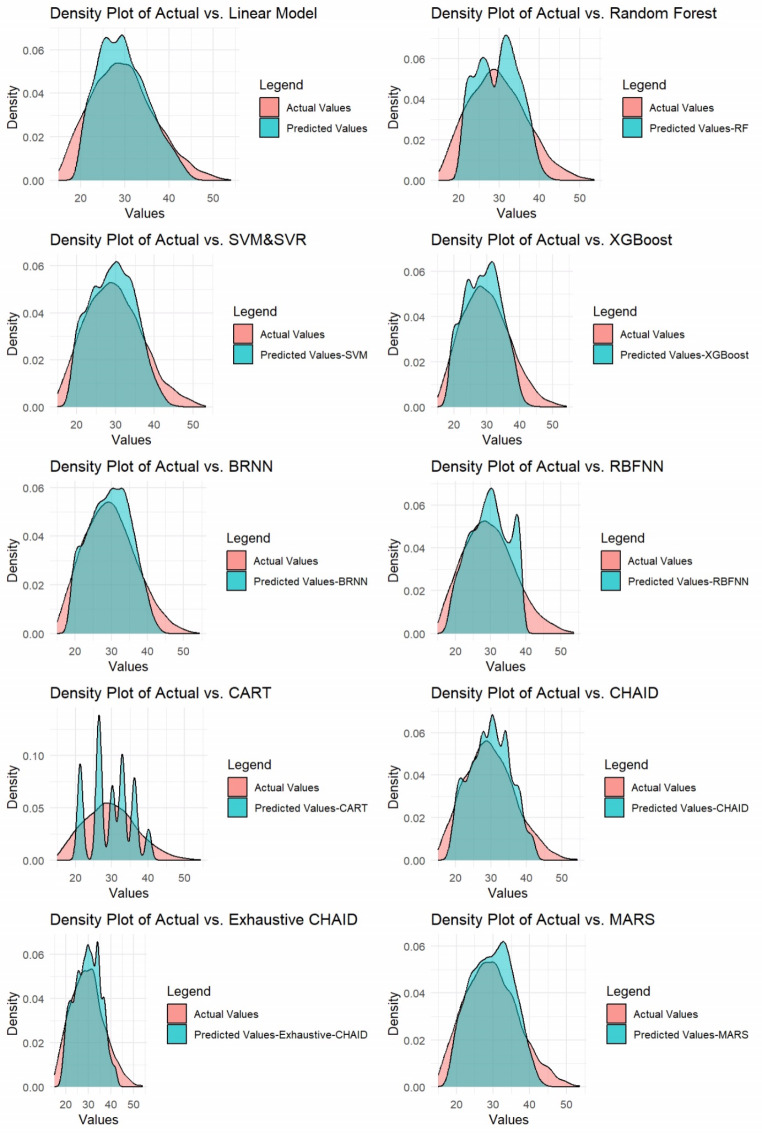
Comparison of actual and predicted values using multiple linear regression and machine learning algorithms

Table 3; Fig. 3 demonstrate that machine learning models (except CART and RBFNN) perform better than multiple linear regression metrics, which is consistent with previous studies (Huma and Iqbal 2019; Yucedag 2019). This highlights the ability of ANN analyses to capture complex and nonlinear relationships, setting it apart from traditional methods like regression analysis. Linear regression assumes a linear relationship between variables (Kebede and Gebretsadik 2010), making it ineffective at modeling nonlinear relationships. Additionally, classical methods like multiple linear regression have various assumptions, including the selection of variables and the fulfillment of the error term assumption, which can affect the reliability of predictive outcomes when not met (Eyduran et al. 2019; Zaborski et al. 2019; Coşkun et al. 2023; Tırınk et al. 2023b).

Ali et al. (2015), conducted a similar study to predict the body weight of Harnai sheep based on their biometric features, employing CART, CHAID, and Exhaustive CHAID algorithms. The ranking of success was as follows: Exhaustive CHAID, CHAID, and CART algorithms. This ranking aligns with our findings, despite the utilization of different sheep breeds and variables in our study. Çelik et al. (2017), conducted a study on the biometric data of Mengali rams. They used various algorithms and found that the predictive accuracy ranking was as follows: CART > CHAID ≈ Exhaustive CHAID > MARS > RBFNN. Our study also found that MARS algorithms performed better than Exhaustive CHAID and CHAID algorithm. However, contrary to these results, in our current study, the CART algorithm was found to be the least successful model, which differed from their findings. Additionally, it was observed that the RBFNN algorithm outperformed the Exhaustive CHAID, CHAID, and CART algorithms, which is an important finding. Huma and Iqbal (2019) conducted a study using biometric data from Balochi breed rams in Pakistan. Their research found that the success ranking of the algorithms used was RF > SVM > Multiple Linear Regression > CART. Interestingly, our study produced similar results despite using different variables and sheep breeds. Olfaz et al. (2019) employed a dataset comprising 366 records from the Karayaka sheep breed, utilizing similar variables to the current study. The CHAID algorithms in this study revealed that the weaning weight was statistically influenced by measurement time, gender, and farm type. Furthermore, the CART algorithm generated a tree diagram, indicating that lambing type had an impact on weaning weight and that the measurement time for single-born lambs was influenced by the lambing type. The conclusion drawn by Olfaz et al. (2019) that the CHAID algorithm is biologically more advantageous than the CART algorithm aligns with the findings of the current study. Faraz et al. (2023) conducted a study employing biometric measurements of indigenous Thalli sheep from Pakistan to predict body weight using CART and MARS algorithms. Consistent with the present investigation, the findings indicated that the MARS algorithm exhibited superior compatibility in contrast to the CART algorithm. Tırınk (2022) utilized biometric measurements of 270 Thalli breed sheep to predict their body weight. Their study ranked the success of algorithms as MARS > BRNN > SVR > RF. This inconsistency with current study can be attributed to differences in independent variables and dataset sizes between the two studies. Tırınk et al. (2023b) used biometric data from 344 Polish Merino sheep to predict weight. This study employed RF, SVR, and CART algorithms based on their success ranking. Our findings align with the results of their study. Tırınk et al. (2023a) conducted a study using data from 393 Romane sheep, similar to our study’s variables, to predict live weight. They ranked the success of their employed algorithms as CART > MARS and SVR. Our findings and results were contradictory to them. Faraz et al. (2023) utilized biometric data from 152 Kajli sheep to predict their weight. The study compared the effectiveness of XGBoost and MARS algorithms, with XGBoost outperforming MARS. Our study aligns with the recommendation of using XGBoost as the preferred algorithm, as found in Faraz et al. (2023).

When the current study is compared with past research results and the chosen criteria for suitability are examined, it becomes evident that the models employed in this study generally yield similar outcomes. The inconsistencies observed across these studies are thought to primarily stem from the variations in species, gender, and the employed variables within the datasets.

## Conclusion

The results of this study demonstrate the potential of Artificial Neural Networks (ANNs) in predicting the live weights of Akkaraman sheep. The Random Forest algorithm was found to be the most successful method for predicting the post-weaning weights of Akkaraman sheep. However, it is important to note that the success of the algorithm may vary depending on the choice of variables and breed factors.

This study also highlights the importance of utilizing both machine learning algorithms and classical regression methods in predicting body weights based on explanatory variables. By combining these methods, more accurate models can be constructed, which can benefit breeders and researchers in the sheep industry.

Future studies can improve the accuracy of live weight prediction models by exploring alternative algorithms and incorporating additional variables, such as environmental and nutritional factors. Overall, this study provides valuable insights into the use of ANNs for live weight prediction in sheep and opens opportunities for further research in this field.

## Electronic supplementary material

Below is the link to the electronic supplementary material.

Supplementary Material 1

## Data Availability

Data will be provided by the corresponding author on reasonable request.
