# Supplementary material for: Comparison of machine learning algorithms and multiple linear regression for live weight estimation of Akkaraman lambs
Source: Trop Anim Health Prod. 2024 Sep 3;56(7):250. doi: 10.1007/s11250-024-04049-0 (PMC11371844; doi:10.1007/s11250-024-04049-0)
Supplement: Supplementary file 1 — Supplementary Material 1 [file 11250_2024_4049_MOESM1_ESM.pdf]

References

Ali, M., Eyduran, E., Tariq, M. M., Tirink, C., Abbas, F., Bajwa, M. A., Baloch, M. H., Nizamani, A. H., Waheed, A., Awan, M. A., Shah, S. H., Ahmad, Z. and Jan, S. 2015. Comparison of artificial neural network and decision tree algorithms used for predicting live weight at post-weaning period from some biometrical characteristics in Harnai sheep. Pakistan Journal of Zoology, 47(6), 1579-1585. https://doi.org/0030-9923/2015/0006-1579.

Ananthaneni, Y., Teja, A. C. and Meena, S. D. 2023. Sustain our variable populace through trenchant-edge carur-manufacturing and agriculture innovation. In AIP Conference Proceedings. 27 October, Chittoor, India.

Biggs, D., De Ville, B. and Suen, E. 1991. A method of choosing multiway partitions for classification and decision trees. Journal of Applied Statistics, 18, 49-62. https://doi.org/10.1080/02664769100000005.

Boujenane, I., and Halhaly, S. 2015. Estimation of body weight from heart girth in Sardi and Timahdite sheep using different models. Iranian Journal of Applied Animal Science, 5(3), 639-646.

Breiman, L. 2017. Classification and Regression Trees. In Editor LB. Taylor and Francis Group. 6th ed., 368, New York, USA.

Canaza-Cayo, A. W., Churata-Huacani, R., Çakmakçı, C., Rodríguez-Huanca, F. H., de Sousa Bueno Filho, J. S., Fernandes, T. J., and De La Cruz, Y. C. R. 2024. Use of machine learning approaches for body weight prediction in Peruvian Corriedale Sheep. Smart Agricultural Technology, 100419.

Çelik, S., Eyduran, E., Karadas, K. and Tariq, M. M. 2017. Comparison of predictive performance of data mining algorithms in predicting body weight in Mengali rams of Pakistan. Revista Brasileira de Zootecnia, 46(11), 863–872. https://doi.org/10.1590/S1806-92902017001100005.

Chen, T. and Guestrin, C. 2016. Xgboost. A scalable tree boosting system. In KDD 16: Proceedings of the 22nd ACM SIGKDD International Conference on Knowledge Discovery and Data Mining. 13-17 August, San Francisco, California, USA.

Cilgin, C., Goksen, Y., and Goksen, H. 2023. The Effect of Outlier Detection Methods in Real Estate Valuation with Machine Learning. İzmir Journal of Social Sciences, 5(1).

Coşkun, G., Şahin, Ö., Altay, Y. and Aytekin, İ. 2023. Final fattening live weight prediction in Anatolian Merinos lambs from some body characteristics at the initial of fattening by using some data mining algorithms. Black Sea Journal of Agriculture, 6(1), 47-53. https://doi.org/10.47115/bsagriculture.1181444.

Duguma, G., Mirkena, T., Haile, A., Iñiguez, L., Okeyo, A. M., Tibbo, M., Rischkowsky, B., Sölkner, J. and Wurzinger, M. 2010. Participatory approaches to investigate breeding objectives of livestock keepers.

Participatory definition of breeding objectives and implementation of community-based sheep breeding programs in Ethiopia. PhD Thesis. University of Natural Resources and Life Sciences, Department of Sustainable Agricultural Systems, Vienna, Austria.

Eksteen, S. and Breetzke, G. D. 2011. Predicting the abundance of African horse sickness vectors in South Africa using GIS and artificial neural networks. South African Journal of Science, 107(7), 1-8. https://doi.org/10.4102/sajs.v107i7/8.404.

Erol, R., Oğulata, S. N., Şahin, C. and Alparslan, Z. N. 2008. A radial basis function neural network (RBFNN) approach for structural classification of thyroid diseases. Journal of Medical Systems, 32, 215-220. https://doi.org/10.1007/s10916-007-9125-5.

Eyduran, E., Akin, M. and Eyduran, S. P. 2019. Application of Multivariate Adaptive Regression Splines through R Software. 1st ed. 112, Nobel Academic Publishing, Ankara.

FAO. 2023. Food and Agriculture Organization (FAO) of the United Nations. Livestock Primary. Food and Agriculture Organization of the United Nations. https://www.fao.org/faostat/en/#data/QCL. Accessed 21 July 2023.

Faraz, A., Tırınk, C., Önder, H., Şen, U., Ishaq, H. M., Tauqir, N. A., Waheed, A. and Nabeel, M. S. 2023. Usage of the XGBoost and MARS algorithms for predicting body weight in Kajli sheep breed. Tropical Animal Health and Production, 55(4), 276. https://doi.org/10.1007/s11250-023-03700-6.

Friedman, J. H. 1991. Multivariate adaptive regression splines. Annals of Statistics, 19(1), 1-67. https://doi.org/10.1214/aos/1176347963.

Friedman, J. H. 2001. Greedy function approximation: a gradient boosting machine. Annals of Statistics, 29(5), 1189-1232.

Fukuda, O., Nabeoka, N. and Miyajima, T. 2013. Estimation of marbling score in live cattle based on ICA and a neural network. International Conference on Systems, Man and Cybernetics, 13-16 October, Manchester, UK. https://doi.org/10.1109/SMC.2013.280.

Gilbert, R. C., Richman, M. B., Trafalis, T. B. and Leslie, L. M. 2010. Machine learning methods for data assimilation. Computational Intelligence in Architecturing Complex Engineering Systems, 105-112.

Gilbert, R. C., Richman, M. B., Trafalis, T. B., and Leslie, L. M. (2010). Machine learning methods for data assimilation. Computational Intelligence in Architecturing Complex Engineering Systems, 105-112.

Hamadani, A. and Ganai, N. 2023. Evaluation and ranking of artificial intelligence algorithms for performance prediction in sheep. Scientific Reports, 13, 13242. https://doi.org/10.1038/s41598-023-40528-4.

Hamadani, A., Ganai, N. A., Mudasir, S., Shanaz, S., Alam, S. and Hussain, I. 2022. Comparison of artificial intelligence algorithms and their ranking for the prediction of genetic merit in sheep. Scientific Reports, 12(1), 18726. https://doi.org/10.1038/s41598-022-23499.

Huma, Z. E. and Iqbal, F. 2019. Predicting the body weight of Balochi sheep using a machine learning approach. Turkish Journal of Veterinary & Animal Sciences, 43(4), 500-506. https://doi.org/10.3906/vet-1812-23.

Iqbal, F., Waheed, A. and Faraz, A. 2022. Comparing the predictive ability of machine learning methods in predicting the live body weight of Beetal Goats of Pakistan. Pakistan Journal of Zoology, 54(1), 231–238. https://doi.org/10.17582/journal.pjz/20191003081007.

Karadas, K., Tariq, M., Tariq, M. M. and Eyduran, E. 2017. Measuring Predictive Performance of Data Mining and Artificial Neural Network Algorithms for Predicting Lactation Milk Yield in Indigenous Akkaraman Sheep. Pakistan Journal of Zoology, 49(1), 1-7. https://doi.org/10.17582/journal.pjz/2017.49.1.1.7.

Kass, G. V. 1980. An exploratory technique for investigating large quantities of categorical data. Journal of Applied Statistics, 29(2), 119-127. https://doi.org/10.2307/2986296.

Kebede, K. and Gebretsadik, G., 2010. Statistical modelling of growth performance data on sheep using mixed linear models. Livestock Research for Rural Development. 22(4), Article #80. http://www.lrrd.org/lrrd22/4/kefe22080.htm.

Koc, Y., Eyduran, E., and Akbulut, O. 2017. Application of regression tree method for different data from animal science. Pakistan Journal of Zoology, 49(2), 599-607. https://doi.org/10.17582/journal.pjz/2017.49.2.599.607.

LeCun, Y., Bengio, Y., and Hinton, G. 2015. Deep learning. Nature, 521, 436-444. https://doi.org/10.1038/nature14539.

Neethirajan, S. 2020. The role of sensors, big data and machine learning in modern animal farming. Sens. Sensing and Bio-Sensing Research, 29, 100367. https://doi.org/10.1016/j.sbsr.2020.100367.

Olfaz, M., Tırınk, C. and Önder, H. 2019. Use of CART and CHAID algorithms in Karayaka sheep breeding. Journal of Kafkas University Veterinary Faculty, 25(1), 105-110. https://doi.org/10.9775/kvfd.2018.20388.

Pérez-Rodríguez, P., Gianola, D., Weigel, K. A., Rosa, G. J. M., and Crossa, J. 2013. An R package for fitting Bayesian regularized neural networks with applications in animal breeding. Journal of Animal Science, 91(8), 3522-3531. https://doi.org/10.2527/jas.2012-6162.

R Studio Team. 2023. RStudio: Integrated Development Environment for R. R Studio, PBC, Boston, MA. URL http://www.rstudio.com/; Accessed 13 July 2023

Samperio, E., Lidón, I., Rebollar, R., Castejón-Limas, M., & Álvarez-Aparicio, C. (2021). Lambs' live weight estimation using 3D images. Animal, 15(5), 100212.

Sen, P. C., Hajra, M., and Ghosh, M. 2018. Supervised classification algorithms in machine learning: A survey and review. In Emerging Technology in Modelling and Graphics: Proceedings of IEM Graph 2018 (pp. 99-111). Springer Singapore.

Tırınk, C. 2022. Comparison of Bayesian Regularized Neural Network, Random Forest Regression, Support Vector Regression and Multivariate Adaptive Regression Splines Algorithms to predict body weight from biometrical measurements in Thalli Sheep. Journal of Kafkas University Veterinary Faculty, 28(3), 411–419. https://doi.org/10.9775/kvfd.2022.27164.

Tırınk, C., Önder, H., Francois, D., Marcon, D., Şen, U., Shaikenova, K., Omarova, K., and Tyasi, T. L. 2023a. Comparison of the data mining and machine learning algorithms for predicting the final body weight for Romane sheep breed. PLoS ONE, 18(8), e0289348. https://doi.org/10.1371/journal.pone.0289348.

Tırınk, C., Piwczyński, D., Kolenda, M., and Önder, H. 2023b. Estimation of body weight based on biometric measurements by using random forest regression, support vector regression and CART algorithms. Animals, 13(5), 798. https://doi.org/10.3390/ani13050798.

Vapnik, V. N. 2000. The Nature of Statistical Learning Theory. 2nd Edition, Springer, Berlin. https://doi.org/10.1007/978-1-4757-3264-1.

Vapnik, V., Golowich, S., and Smola, A. 1996. Support vector method for function approximation, regression estimation and signal processing. NIPS, 9, 281-287.

Yang, X. Z., Lacroix, R., and Wade, K. M. 2000. Investigation into the production and conformation traits associated with clinical mastitis using artificial neural networks. Canadian Journal of Animal Science, 80(3), 415-426. https://doi.org/10.4141/A98-100.

Younas, U., Abdullah, M., Bhatti, J. A., Pasha, T. N., Ahmad, N., Nasir, M. and Hussain, A. J. J. A. P. S. 2013. Inter-relationship of body weight with linear body measurements in Hissardale sheep at different stages of life. The Journal of Animal & Plant Sciences, 23(1), 40-44.

Yucedag, N. 2019. İvesi koyunlarında laktasyon süt verimlerinin çoklu doğrusal regresyon ve yapay sinir ağı modelleriyle karşılaştırılmalı incelenmesi. Master's Thesis. Ankara University, Institute of Science, Ankara, Türkiye.

Zaborski, D., Ali, M., Eyduran, E., Grzesiak, W., Tariq, M. M., Abbas, F., Waheed, A., and Tırınk, C. 2019. Prediction of selected reproductive traits of indigenous Harnai sheep under the farm management system

via various data mining algorithms. Pakistan Journal of Zoology, 51(2), 421-431. https://doi.org/10.17582/journal.pjz/2019.51.2.421.431.

Zaborski, D., and Grzesiak, W. 2011. Detection of difficult calvings in dairy cows using neural classifier. Archives Animal Breeding, 54(5), 477-489. https://doi.org/10.5194/aab-54-477-2011.

Zhang, G., and Hu, M. Y. 1998. Neural Network Forecasting of the British Pound/US Dollar Exchange Rate. Omega, Int Journal of Management and Science, 26(4), 495-506. https://doi.org/10.1016/S0305-0483(98)00003-6.
